# *Theileria orientalis* Ikeda infection does not negatively impact growth performance or breeding soundness exam results in young beef bulls at bull test stations

**DOI:** 10.3389/fvets.2024.1432228

**Published:** 2024-07-18

**Authors:** Sierra R. Guynn, Scott P. Greiner, John F. Currin, S. Michelle Todd, Alphonce Assenga, Laura L. Hungerford, Kevin K. Lahmers

**Affiliations:** ^1^Department of Biomedical Sciences and Pathobiology, Virginia-Maryland College of Veterinary Medicine, Virginia Polytechnic Institute and State University, Blacksburg, VA, United States; ^2^School of Animal Sciences, Virginia Tech, Blacksburg, VA, United States; ^3^Department of Large Animal Clinical Sciences, Virginia-Maryland College of Veterinary Medicine, Virginia Polytechnic Institute and State University, Blacksburg, VA, United States; ^4^Department of Population Health Sciences, Virginia-Maryland College of Veterinary Medicine, Virginia Polytechnic Institute and State University, Blacksburg, VA, United States

**Keywords:** beef cattle, *Theileria orientalis* (Ikeda), average daily gain (ADG), breeding soundness evaluation (BSE), bull

## Abstract

**Introduction:**

*Theileria orientalis* Ikeda genotype is an emerging cattle disease in the US. Since 2017, when *T. orientalis* Ikeda was discovered in beef cattle in two counties in Virginia, cattle infections have risen to include ~67% of Virginia counties and 14 states. Consistent with New Zealand studies, many infected herds in Virginia were >90% positive upon initial testing without overt evidence of infection. Central bull tests present a unique opportunity to study the effects of *T. orientalis* Ikeda infections, as bulls from multiple source herds are consolidated. The objective of this study was to determine if infection with *T. orientalis* Ikeda affected the average daily gain (ADG), adjusted yearling weight (AYW) and breeding soundness of bulls at two test stations in Virginia over a period of years.

**Materials and methods:**

The bulls were fed and housed similarly to compare their growth performance and breeding soundness. For *T. orientalis* Ikeda testing, DNA was extracted from whole blood for quantitative polymerase chain reaction.

**Results:**

The number of bulls infected with *T. orientalis* Ikeda at initial delivery to the stations increased significantly over the years studied. Multivariable linear regression models, using Angus bulls from Virginia test stations, indicated no significant effect on ADG or AYW in bulls that became test positive during the test or were positive for the duration, compared to Angus bulls that were negative for the duration. At LOC A, the odds of passing a breeding soundness exam (BSE) were not significantly different for bulls that turned positive during the test or were positive for the duration, compared to bulls that were negative for the duration of the test. At LOC B, bulls that became positive during the test were 2.4 times more likely (95% CI: 1.165–4.995, *p* = 0.016) to pass their BSE compared to bulls that remained negative throughout the test.

**Discussion:**

We do not suppose that an obscured infection of *T. orientalis* Ikeda is protective for bulls to pass a BSE. However, this study demonstrates an obscured infection of *T. orientalis* Ikeda does not negatively affect weight gain or achievement of a satisfactory BSE rating at the central bull test stations in Virginia.

## 1 Introduction

In 2017, *Theileria orientalis* genotype Ikeda was first detected in the United States (US) in beef cattle from multiple counties in Virginia (1). In Virginia between 2019 and 2021, surveillance testing of market cattle for *T. orientalis* Ikeda, increased from <2 to ~35% of samples testing positive in the Northern and Southwest markets with an overall prevalence of 8.7% in 2021 (2). This emerging cattle disease has since been discovered in 14 states as of November 2023 (3). *T. orientalis* is a tick-borne non-transforming hemoprotozoan (4) with 11 genotypes as determined by molecular characterizations of the major piroplasm surface protein (MPSP) (5). Of the 11 genotypes of *T. orientalis*, type 2 (Ikeda) is meaningfully associated with clinical disease in Australia, New Zealand, Japan (4) and the US (1).

The primary biologic vector of *T. orientalis* Ikeda in New Zealand, Japan (4) and the US is *Haemaphysalis longicornis*, the Longhorned tick (LT) (6, 7). As of March 2024, the LT has been found in 19 states (8). The biology of the LT includes parthenogenetic reproduction, aggressive biting, minimal host specificity, and swarming behavior which allows for rapid increases in both population numbers and infested territories (9, 10). This biology, and that the LT spends the majority of its lifecycle off the host in the environment, makes LT control measures problematic (11). The expanding distribution of the LT has followed predictions in the Eastern US (12, 13), while expanding farther west, north and south than expected. With the geographic distribution of *T. orientalis* Ikeda closely mirroring that of the LT, spread of *T. orientalis* Ikeda to non-endemic regions through expansion of the LT distribution must be anticipated.

Acute clinical infection from *T. orientalis* Ikeda can cause anemia, icterus, ill-thrift, abortions and death in naïve cattle particularly if they are stressed due to parturition, transportation or a significant change in husbandry (1, 4, 14). A sequela of *T. orientalis* Ikeda infection is the cattle become asymptomatic carriers, potentially for life (15). However, in other countries, positive beef herds without overt clinical signs have been described and associated with decreased live weight gain in weaned beef calves (16) and decreased mean daily gain in suckling beef calves (17). It was thought the persistently infected cattle could recrudesce into clinical disease particularly under times of stress, however it does not appear to frequently occur (18). However, theileriosis, even if apparently asymptomatic or in the persistently infected chronic phase, “decreases the fitness of the animal to tolerate other endemic diseases and deficiencies” (19). Additionally, there are no available treatments or vaccines for *T. orientalis* in the US, and only one effective treatment available elsewhere requiring extended meat withdrawal and cost (4). As the LT and cattle theileriosis spread in the US, creating endemicity, it will be crucial to understand the effects of *T. orientalis* Ikeda infections on beef production parameters such as growth performance, reproductive soundness and longevity within the herd.

The Virginia Beef Cattle Improvement Association has operated bull performance test stations for over 60 years, and currently operates two separate stations annually. Bull test stations provide bull producers an opportunity to compare their bull's growth performance to other bulls in a controlled environment, culminating in a value-added sale based upon the bull's performance. Bull test stations provide an excellent opportunity to study the effects of *T. orientalis* Ikeda infection on weight gain, as feed and environment are the same for all the bulls at each location. Another advantage of the test stations is that the entry requirements provide a generally homogenous population of bulls with respect to age and health status to study reproductive soundness.

The purpose of this retrospective study was to determine if weight gain or reproductive soundness of bulls at Virginia bull test stations was affected by the bulls' *T. orientalis* Ikeda status. Our hypothesis was that those bulls that were negative for *T. orientalis* Ikeda for the duration of the bull test would perform better with respect to gain and reproductive soundness compared to bulls that were positive for the whole duration or became positive during the bull test.

## 2 Materials and methods

### 2.1 Bull management and growth data

The bull test stations were operated at two locations in Virginia by the Virginia Beef Cattle Improvement Association. At LOC A (in Louisa County), fall born bulls (SR) were delivered to the test facility in Late June at 8–10 months of age. At LOC B (in Wythe County), both the fall born (SR) and spring-born (JR) bulls were delivered to the test facility in early October at 10–12 and 7–9 months of age respectively. At both stations, growth performance was evaluated over a 112-day period with qualifying bulls sold at a special sale ~6 weeks later. For this study, bulls were placed on the study at the time of delivery to their respective station until BSE's were completed after the growth performance test, which was for ~5 months. All bulls originated from cooperating farms in Virginia or bordering states.

At delivery, bulls were accompanied by a Certificate of Veterinary Inspection, tested negative to anaplasmosis and bovine viral diarrhea (BVD) persistent infection and met post-weaning management and vaccination requirements. Requirements included a 7-strain Clostridial, *Pasteurella*, and a modified live 5-way respiratory viral vaccination. At delivery, bulls were examined for reproductive soundness by measuring testicular circumference and unsound bulls were eliminated. Bulls received a 7-strain Clostridium booster, 5-way respiratory virus booster, and were treated for internal and external parasites with pour-on Cydectin^®^ (Elanco Animal Health, Greenfield IN; 5 mg/ml moxidectin, dose 0.5 mg/kg topically) at delivery. Lastly, at delivery, all bulls were bled via the caudal tail vein into an EDTA anticoagulant tube for molecular diagnostics and testing for *T. orientalis* Ikeda as described below. Only single use needles were used for all injections. The blood sampling was reviewed and approved by the Institutional Animal Care and Use Committee of Virginia Tech (protocol #21-248).

Each bull's attitude and appearance were examined daily, however unless they appeared ill or anorexic, they were only put the through the chute system periodically for weighing. At LOC A, bulls were housed in 12-acre grass lots with the woods on the edges mostly fenced out, in groups of ~50 according to breed, age, and weight. At LOC B, bulls were housed in 12–30-acre grass lots that contained some woods, but grouped similarly. At both locations, a corn-silage based total mixed ration was fed with a target average daily gain (ADG) of 1.6 kg/day, formulated using the National Research Council Nutrient Requirements of Beef Cattle (20). Following a 14–21-day diet acclimation period, weight gain was measured over a 112-day period. Average daily gain (ADG) and adjusted yearling weight (AYW) were calculated along with ratios for animals of the same breed and age (SR vs. JR) according to Guidelines for Uniform Beef Improvement Programs standards (21). Because the LOC B test occurred over winter, the bulls were also treated for external parasites in early January. At completion of the test every bull's scrotal circumference was measured, caudal tail vein blood was obtained for *T. orientalis* Ikeda testing, and SR bulls had a breeding soundness exam (BSE) performed. Approximately 10%−20% of SR bulls did not receive a BSE as they were not sale eligible based on poor test growth performance, structural unsoundness, illness or other issue.

Both bull tests consisted of a 2–3-week transition period, 112 days on feed performance testing, and then an ~6-week period until the sale of the bulls. At the end of the feed performance test, a complete breeding soundness exam (BSE) is performed on sale-eligible bulls. *T. orientalis* Ikeda was first detected in 2019 when the bulls arrived at the southwest Virginia bull test station, but testing was not conducted on the second location until 2021. An acute case of *T. orientalis* Ikeda with clinical signs severe enough to require pulling a bull from its group, for exam by bull station staff or a veterinarian, was not noted at either test station to date.

### 2.2 Breeding soundness exam

Components of the BSE for SR bulls included a brief physical exam, rectal palpation of the accessory sex glands, palpation of the testis, determination of scrotal circumference, and electroejaculation. Electroejaculation utilized the same program for each bull via the Pulsator IV (prior to 2022) and the Pulsator V (after 2022) (Lane Manufacturing, Denver CO). Semen motility was evaluated by wet mount chute side with 30% progressive motility required for a satisfactory rating. Morphology was determined using Eosin-stained slides examined under oil immersion at 100X with 70% normal sperm required for a satisfactory rating. If a bull was deferred at the initial BSE that occurred at the end of the growth performance test, a second BSE was performed ~3 weeks later for final determination of BSE result. For the purposes of this study, a bull was graded either “not-eligible,” satisfactory or unsatisfactory.

### 2.3 Molecular diagnostics

DNA was extracted from K_2_-EDTA anticoagulated blood (DNeasy blood and tissue kit; Qiagen) following the manufacturer's protocol using pre-warmed (56°C elution buffer. The initial blood volume for DNA extraction was 100 μl. We used a duplex TaqMan-based rtPCR assay to detect the major surface protein 1b (msp1b) gene of *Anaplasma marginale* and the major piroplasm surface protein (MPSP) gene of *T. orientalis* as described by our lab previously (22). The primers and probes for MPSP are specific for *T. orientalis* but are not genotype-specific. To further characterize genotype a second multiplex rtPCR assay targeting Ikeda, Chitose, and Buffeli genotypes as described by our lab previously (22).

Each year, the bulls were tested for *T. orientalis* Ikeda twice; at delivery and at the end of the feed performance test when BSEs were performed on eligible bulls. Based upon the *T. orientalis* Ikeda results from the start of the test to the end of the feed performance test, bulls were classified as either negative throughout the test (NN), positive throughout the test (PP), or negative on intake and positive at the end of the test (NP). There are two scenarios where a bull could be classified as a NP bull; they were infected shortly before delivery and did not have enough time to become test positive at delivery sampling or they became positive while at the bull test station.

### 2.4 Statistical analysis

Data were entered into spreadsheets (Excel; Microsoft Corp.) and subsequently analyzed using JMP^®^ Pro (JMP^®^, Version 16. SAS Institute Inc., Cary, NC 1989–2013). Prevalence (95% CI) of bulls positive at intake to the test station were compared for each location by year and tested with a Fisher's exact test at LOC A or Cochran-Armitage test for trend at LOC B. There were three distinct populations of bulls from the two stations; LOC A all SR bulls (2021–2022), LOC B SR bulls (2020–2023) and LOC B JR bulls (2020–2023). Angus was the majority breed in all three populations of bulls with too few bulls of other breeds for analysis, so analyses for *average daily gain* (ADG) and adjusted yearling weight (AYW) were restricted to purebred Angus bull data due to well documented breed differences expected with gain (23–25). Means for ADG and AYW were determined with 95% confidence intervals (CI). A one-way ANOVA with Dunnets method for means comparison was used to compare the NP and PP bulls to the control group NN. In multivariable linear regression models for ADG and AYW, *T. orientalis* Ikeda status was forced into each model and other potential covariables included year of test, age and weight at delivery, group within test, and state of origin. The cut-off for potential covariates to be included in the final models was *p* < 0.05. Then using the Dunnets method, within the model, the *T. orientalis* Ikeda statuses of NP and PP were compared to the control group NN. To assess effects of infection on eligibility for a BSE, the NP and PP bulls were separately compared to the NN group to determine the ratio of success between the groups, with the 95% CIs and Fisher's exact test. In multivariable logistic regression models for BSE eligibility, *T. orientalis* Ikeda status was forced into each model and other potential covariables included year of test, ADG ratio, and AYW ratio. The ADG and AYW ratios were used in the BSE models because all breeds were represented in the BSE data, and the ratios normalized the breed differences in gain. To assess effects of infection on achieving a satisfactory rating in the BSE, the NP and PP bulls were separately compared to the NN group to determine the ratio of successes between groups, with the 95% CIs and Fisher's exact test. In multivariable logistic regression models for BSE success, *T. orientalis* Ikeda status was forced into each model. We used forward stepwise multivariable logistic regression analyses with Bayesian Information Criteria as the stopping rule and *p* < 0.05 as the cut-off for potential covariates: year, state of origin, pen number, breed, bull conception type, age at delivery, weight at delivery, ADG and AYW.

## 3 Results

### 3.1 Bull test station populations

A total of 584 senior and 416 junior bulls were examined in this study (Table 1). For all three populations the majority of the bulls were purebred Angus, though there was representation from many different breeds. At LOC A, ~92% of the bulls were from Virginia with <8% of bulls from West Virginia and North Carolina. At LOC B for both JR and SR groups, ~62% of the bulls were from Virginia. Approximately 23% of the LOC B bulls were from Tennessee, while <15% of the LOC B bulls were from West Virginia and North Carolina. For the two SR populations of bulls ~68% of the bulls passed a BSE with a satisfactory rating. JR bulls were too young for a BSE. The three populations of purebred Angus bulls used in the growth performance analyses are described in Table 2.

**Table 1:** Descriptive statistics of the three populations of bulls including all breeds; LOC A (years 2021–2022), LOC B seniors (years 2020–2023) and LOC B juniors (years 2020–2023).

		**LOC A *n* (%)**	**LOC B Seniors *n* (%)**	**LOC B Juniors *n* (%)**
Total bulls		265	361	451
Total with complete sampling data		253 (95)	331 (92)	416 (92)
*Theileria orientalis* status	Negative → negative (NN)	71 (28.1)	129 (39.0)	195 (46.9)
	Negative → positive (NP)	46 (18.2)	102 (30.8)	56 (14.2)
	Positive → positive (PP)	136 (53.7)	100 (30.2)	162 (38.9)
BSE results	Satisfactory	174 (68.8)	226 (68.3)	
	Unsatisfactory	20 (7.9)	59 (17.8)	
	Not eligible	59 (23.3)	46 (13.9)	

**Table 2:** Descriptive statistics of the Angus bulls within each subpopulation from the Virginia bull test stations, for the variables used in the multivariable models of average daily gain and adjusted yearling weight.

		**LOC A, 170 angus**	**LOC B seniors, 169 angus**	**LOC B juniors, 172 angus**
Average daily gain, kg/day (mean ± SD)		1.680 ± 0.251	1.661 ± 0.495	1.506 ± 0.293
Adjusted yearling weight adjusted, kg (mean ± SD)		524.60 ± 37.97	482.95 ± 43.99	517.72 ± 46.07
Weight at delivery, kg (mean ± SD)		364.82 ± 41.32	451.88 ± 61.90	328.75 ± 47.13
Age at delivery, day (mean ± SD)		280.92 ± 23.25	374.25 + 34.62	255.64 ± 22.05
*Theileria orientalis* status	NN, *n* (%)	52 (30.6)	72 (42.6)	88 (51.2)
	NP, *n* (%)	34 (20)	61 (36.1)	25 (14.5)
	PP, *n* (%)	84 (49.4)	36 (21.3)	59 (34.3)

### 3.2 *Theileria orientalis* Ikeda testing

**Figure 1:**
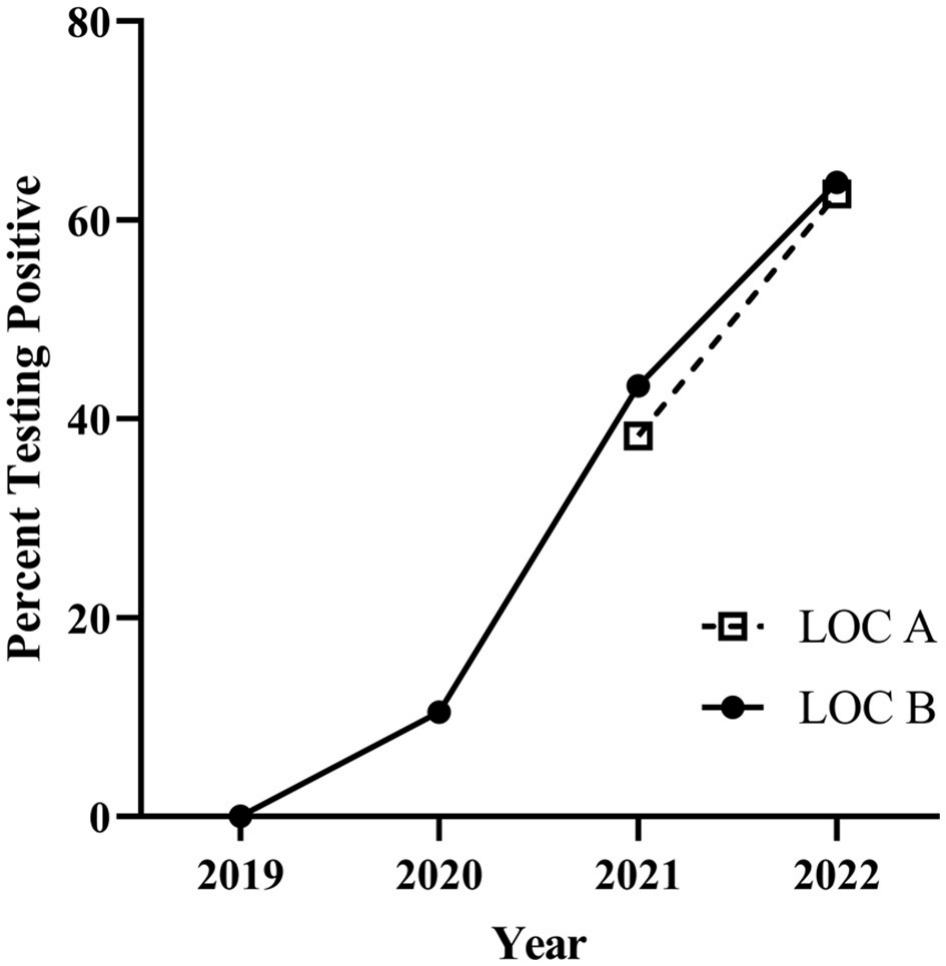
*Theileria orientalis* Ikeda percentage of positive bulls on intake to the bull test stations in Virginia for years indicated. LOC A–2021 40.3% (31.8–49.5 95% CI), 2022 64.7% (56.5–72.2 95% CI). LOC B–2019 0.8% (0.1–4.5 95% CI), 2020 10.4% (6.9–15.4 95% CI), 2021 49.1% (42.5–55.7 95% CI), 2022 64.4% (57.7–70.6 95% CI). CI: 95% confidence interval.

At both Virginia bull test stations, there has been a significant (*p* < 0.0001) increase in the percent of bulls that tested positive to *T. orientalis* Ikeda at delivery over the years tested (Figure 1).

### 3.3 Average daily gain and adjusted yearling weight

With univariate analysis, ADG was significantly higher among NP bulls compared to NN bulls in both the LOC B JR and SR populations and for PP bulls compared to NN bulls among the LOC B SR population (Table 3). After adjusting for year of test, age at delivery and weight at delivery; there were no significant differences regardless of *T. orientalis* Ikeda status.

**Table 3:** Unadjusted and adjusted ADG (kg/day) of the NP and PP bulls, compared to NN bulls, within the three populations at the Virginia bull test stations for the years indicated.

**Ikeda status**	**Model**	**ADG, kg/day**	**95% Confidence interval**	***p*-value**
**LOC A–2021 and 2022**				
NN	Univariate	1.708	1.640–1.777	Reference
NN	Multivariable	1.694	1.658–1.730	Reference
NP	Univariate	1.674	1.569–1.739	0.646
NP	Multivariable	1.663	1.618–1.708	0.461
PP	Univariate	1.654	1.619–1.728	0.520
PP	Multivariate	1.675	1.646–1.704	0.648
**LOC B seniors–2020–2023**				
NN	Univariate	1.385	1.285–1.486	Reference
NN	Multivariable	1.623	1.522–1.724	Reference
NP	Univariate	1.934	1.825–2.043	**<0.0001**
NP	Multivariable	1.726	1.335–1.817	0.349
PP	Univariate	1.752	1.611–1.894	**<0.0001**
PP	Multivariable	1.712	1.610–1.815	0.356
**LOC B Juniors–2020–2023**				
NN	Univariate	1.497	1.437–1.557	Reference
NN	Multivariable	1.500	1.456–1.544	Reference
NP	Univariate	1.670	1.557–1.782	**0.017**
NP	Multivariable	1.556	1.464–1.648	0.485
PP	Univariate	1.452	1.378–1.525	0.561
PP	Multivariable	1.506	1.445–1.567	0.956

Multivariable adjusted models control for year of test, weight at delivery, and age at delivery. Values in bold are significant at *p* < 0.01.

In the univariate comparison, LOC B senior bulls with a NP *T. orientalis* Ikeda status had a significantly higher AYW compared to NN bulls (Table 4). However, after adjustment for year of test, age at delivery and weight at delivery, this difference vanished. There were no significant differences in AYW for any comparisons between NN and NP senior bulls in either the LOC B or LOC A (Table 4).

**Table 4:** Unadjusted and adjusted AYW (kg) of the NP and PP bulls, compared to the NN bulls, within the three populations at the Virginia bull test stations for the years indicated.

**Ikeda status**	**Model**	**AYW, kg**	**95% Confidence interval**	***p*-value**
**LOC A–2021 and 2022**				
NN	Univariate	519.21	508.79–529.62	Reference
NN	Multivariable	522.91	518.38–527.43	Reference
NP	Univariate	526.06	513.19–538.94	0.627
NP	Multivariable	526.16	520.54–531.79	0.580
PP	Univariate	527.34	519.15–535.54	0.374
PP	Multivariable	525.79	522.12–529.45	0.534
**LOC B seniors–2020–2023**				
NN	Univariate	470.49	460.63–480.36	Reference
NN	Multivariable	482.62	472.82–492.42	Reference
NP	Univariate	498.75	488.02–509.47	**0.0004**
NP	Multivariable	481.22	472.33–490.11	0.977
PP	Univariate	481.12	467.16–495.07	0.377
PP	Multivariable	480.45	470.50–490.41	0.930
**LOC B juniors–2020–2023**				
NN	Univariate	517.28	507.65–526.91	Reference
NN	Multivariable	515.78	509.03–522.53	Reference
NP	Univariate	534.19	516.12–552.25	0.193
NP	Multivariable	516.57	502.42–530.71	0.994
PP	Univariate	511.39	499.63–523.15	0.682
PP	Multivariable	520.15	510.84–529.47	0.712

Multivariable adjusted models control for year of test, weight at delivery, and age at delivery. Values in bold are significant at *p* < 0.01.

### 3.4 Breeding soundness exam

There was no significant difference in the likelihood of a NN bull being eligible for a BSE as compared to the NP or PP bulls, at either location (*p* > 0.3 for all multivariable models). BSE eligibility was determined in part by gain during the feed test period, therefore it is no surprise that at both locations the ADG ratio (*p* < 0.002) and AYW ratio (*p* < 0.002) were significantly lower if the bull was ineligible for a BSE, regardless of *T. orientalis* Ikeda status or year of test.

Among bulls of different *T. orientalis* Ikeda status, the only significant difference was a higher likelihood for NP bulls to achieve a satisfactory BSE, compared to NN bulls at LOC B (Table 5). None of the tested covariables were found to be significant at LOC A, therefore only univariate results were reported. However, at LOC B year of test was significant (*p* = 0.0005) when comparing NN and NP bulls regardless of *T. orientalis* Ikeda status. And when comparing NN and PP bulls at LOC B, ADG ratio increased significantly (*p* < 0.01) with the odds of achieving a satisfactory rating on the BSE regardless of *T. orientalis* Ikeda status.

**Table 5:** The odds of *Theileria orientalis* Ikeda status NP or PP bull, using the NN bulls as reference, achieving a satisfactory rating in the BSE at the Virginia bull test stations for the years indicated.

**Ikeda status**	**Model**	**Odds ratio**	**95% confidence interval**	***p*-value**
**LOC A–2021 and 2022**				
NP	Univariate	1.0884	0.2431–4.8735	0.999
NP	Multivariable	No significant covariables		
PP	Univariate	0.7908	0.2635–2.3737	0.791
PP	Multivariable	No significant covariables		
**LOC B—Seniors–2020–2023**				
NP	Univariate	2.4123	1.1651–4.9946	**0.012**
NP	Multivariable (year)	2.4411	0.8489–7.0197	0.098
PP	Univariate	1.39	0.7057–2.7364	0.396
PP	Multivariable (ADG ratio)	1.5888	0.7885–3.201	0.195

Multivariable adjusted models control for the covariables in parentheses. Values in bold are significant at *p* < 0.01.

## 4 Discussion

Cattle *T. orientalis* Ikeda infections are spreading and already endemic in some areas where the LT is present in the US. At this time, a true prevalence of Ikeda infection in Virginia cattle is difficult to ascertain as the majority of surveillance sampling has been convenience sampling at Virginia livestock markets (2) or sampling of specific populations of cattle, such as this study. The increasing number of bulls delivered to the test stations infected over the years studied and the number of bulls obtaining a NP status during our study, confirm *T. orientalis* Ikeda is highly infective. Other authors have found that virulence is highly variable (19) due to the presence of multifactorial risk factors, the relationship of which have yet to be elucidated. This study focused on whether infection in young beef bulls negatively impacted bull performance. These overarching effects of infection are pertinent for beef cow/calf producers, as the vast majority use live cover for breeding. We found no significant differences in test ADG or AYW between Angus bulls that were negative for *T. orientalis* Ikeda infection at both the start and end of the test period; those that were positive at the start and end; or those that became infected during the 5–6 months on test. The bulls at the Virginia bull test stations were all beef breed bulls, 7–14 months of age, and they all gained appropriately for the nutrition provided and according to previous literature (25). Similarly, previous work in 2-year-old dairy bulls that were experimentally infected with *T. orientalis* Ikeda, through intravenous injection of infected blood, found no differences in gain, live weight, hematocrit, or temperature between the infected (*n* = 10) and non-infected (*n* = 7) bulls over the 20-week study (26). At day 70 post-infection, a subset (*n* = 4) of the infected bulls started gaining significantly less than the other six bulls of the infected group. However, a reason for this apparent gain difference among infected animals was not identified.

Two other studies possibly indicate younger beef calves may experience reduced gain if infected with *T. orientalis* Ikeda. In Australia, a group of 30 weaned beef calves negative for *T. orientalis* were moved to an area where *T. orientalis* Ikeda was proven to be in both *H. longicornis* ticks and resident cattle (16). By 3 weeks after arrival, all 30 calves were found to be infected with *T. orientalis* Ikeda. The authors calculated average daily live weight gain (ADLG) every 3 weeks and there were two periods of significantly lower gain in the 24 weeks, the second of which did not appear to be related to parasitemia and was considered a period of declining nutrition as it occurred in the middle of winter (16). At 24 weeks the weight of the 30 calves was less than was expected based upon regional benchmarks of ADLG from years prior. The lack of uninfected calves for comparison and the recognized period of poor nutrition makes these findings difficult to interpret. A New Zealand study investigated gain in 123 suckling beef calves on pasture with their dams that were ~2 months old at the start of the study and 5–7 months old at the end (17). The bred dams had been purchased from an area in New Zealand that was endemically stable for *T. orientalis* Ikeda but were not tested prior to the study and were then moved to a new area of sparser tick distribution prior to calving. Calves that became infected during the study, had a lower mean daily gain in the latter half of the study yielding a ~4.5 kg lower live weight at the end of the study. The *T. orientalis* infection status, genetics and milk production of their dams was unknown. For some chronic infectious diseases, negative health and production effects occur during times of stress or less than optimal nutrition (27). Bulls at the Virginia test stations are managed on a high plane of nutrition with an extensive preventive program against other diseases. This may reduce the stresses upon their immune system which would equip the bulls to better manage *T. orientalis* Ikeda parasitemia.

Other studies have used experimental inoculation of infected blood or pasture-based tick-borne transmission with evidence of *T. orientalis* Ikeda infected ticks proven prior to putting cattle in the environment. At both locations of the Virginia bull test stations, no ticks were found on the bulls at delivery or at any processing or weighing event during the test. Additionally, all bulls were treated with an ectoparasiticide upon delivery (and a second time at LOC B), and individual needles are used for all injections throughout the test. A bull that was infected shortly before delivery may have not reached a detectable parasitemia at delivery. And to limit stress and maintain a viable test station, the bulls were not examined frequently for transient fevers or anemia, therefore a mild but acute infection could have been missed. A low environmental burden of ticks creating a minimal number of ticks on the bulls could also have been overlooked with infrequent examinations. A low number of ticks on the bulls or mechanical transmission by hematogenous flies or lice may be responsible for the transmission of the *T. orientalis* Ikeda. Cattle infected by mechanical transfer or smaller tick burdens take longer to turn positive, have a lower parasitemia, experience a mild anemia if any, are unlikely to experience severe symptoms of disease, and could go undetected unless tested regularly (6, 26, 28). This also may have mitigated the negative effects of infection that occurred at the test stations.

With regard to reproductive soundness, there was no significant difference in the odds of the NP or PP bulls achieving a satisfactory rating compared to the NN bulls at LOC A. This is in agreement with a New Zealand study (29) where dairy bulls were serially tested for reproductive soundness following infection and no changes were found in semen quality. At our LOC B bull test, we found that NP bulls were significantly more likely to achieve a satisfactory rating compared to NN bulls. We do not suggest that *T. orientalis* Ikeda infection enhances the reproductive soundness of the NP bulls. However, it is evidence that acquiring a *T. orientalis* Ikeda infection, without having clinical diseases, does not appear to hinder the future reproductive soundness of younger bulls.

This New Zealand study (29), also included serial measurements of libido and found a 2-week period of decreased libido following infection. This was thought to be a manifestation of the mild anemia and anorexia. In the US, the bull BSE does not typically include a measurement of libido (30). In our study the BSE was not serially performed but was only done at the end of the feeding period to ensure reproductive soundness before sale and libido was not assessed. Therefore, we could not assess transient effects on bull reproductive performance.

In a related study, bulls that became clinically ill following infection with *A. marginale*, another cause of clinical bovine infectious anemia, had significantly decreased scrotal circumference and reduced sperm quality for weeks beyond clinical resolution of the disease (31). There is anecdotal evidence from regional herds in Virginia, showing a gap of 5–7 weeks in pregnancies when bulls were acutely infected in the field during the breeding season (Lahmers, K.K. personal communication). Similarly, the Northland Index herd in New Zealand, reported a 47% decrease in successful pregnancies during their 6-week natural breeding season following infection with theileriosis (19). A clinical infection of *T. orientalis* Ikeda during the breeding season, resulting in decreased libido or decreased sperm quality, would explain these results. Acute disease is more likely with a heavy infected tick load because the LT amplifies infective sporonts by the thousands in their salivary glands, which are then deposited into the bovine host when feeding (32, 33).

There is large variability in the herd-level manifestation of clinical theileriosis in both New Zealand and US outbreaks with some herds experiencing up to a 5% mortality rate and others becoming nearly 100% *T. orientalis* Ikeda positive without any signs of clinical disease (1, 3, 19). Whether this variability is due to nutritional status, genetics or method of transmission is unknown at this time (34). However, the results of this study are encouraging in that bulls that are positive for *T. orientalis* Ikeda or acquire the infection without overt clinical signs, continue to grow appropriately and are able to achieve a satisfactory rating on a BSE exam.

## Data availability statement

The raw data supporting the conclusions of this article will be made available by the authors, without undue reservation.

## Ethics statement

The animal studies were approved by Virginia Tech Institutional Animal Care and Use Committee. The studies were conducted in accordance with the local legislation and institutional requirements. Written informed consent was obtained from the owners for the participation of their animals in this study.

## Author contributions

SGu: Conceptualization, Data curation, Formal analysis, Writing – original draft, Writing – review & editing, Investigation. SGr: Methodology, Writing – review & editing, Data curation. JC: Investigation, Writing – review & editing, Methodology. ST: Methodology, Writing – review & editing. AA: Methodology, Writing – review & editing. LH: Methodology, Writing – review & editing, Formal analysis. KL: Funding acquisition, Methodology, Resources, Writing – review & editing.
